# Right Hemihepatectomy by Suprahilar Intrahepatic Transection of the Right Hemipedicle using a Vascular Stapler

**DOI:** 10.3791/1750

**Published:** 2010-01-25

**Authors:** Ingmar Königsrainer, Silvio Nadalin, Alfred Königsrainer

**Affiliations:** Department of General, Visceral, and Transplant Surgery, Tübingen University Hospital

## Abstract

Successful hepatic resection requires profound anatomical knowledge and delicate surgical technique. Hemihepatectomies are mostly performed after preparing the extrahepatic hilar structures within the hepatoduodenal ligament, even in benign tumours or liver metastasis.^1-5^. Regional extrahepatic lymphadenectomy is an oncological standard in hilar cholangiocarcinoma, intrahepatic cholangio-cellular carcinoma and hepatocellular carcinoma, whereas lymph node metastases in the hepatic hilus in patients with liver metastasis are rarely occult. Major disadvantages of these procedures are the complex preparation of the hilus with the risk of injuring contralateral structures and the possibility of bleeding from portal vein side-branches or impaired perfusion of bile ducts. We developed a technique of right hemihepatectomy or resection of the left lateral segments with intrahepatic transection of the pedicle that leaves the hepatoduodenal ligament completely untouched. ^6^ However, if intraoperative visualization or palpation of the ligament is suspicious for tumor infiltration or lymph node metastasis, the hilus should be explored and a lymphadenectomy performed.

**Figure:**
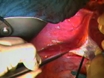


## Protocol

### Surgical Technique

The patient is bedded under standard operative room conditions in dorsal position and the whole abdomen is desinfected. Next, the patient is covered with sterile sheets.
After "reverse L-shaped" laparotomy the abdominal cavity is explored to exclude peritoneal carcinomatosis. Subsequently the ligamentum teres and the ligamentum falciforme are divided with ligatures and electrocautery.
Following exploration of the abdominal cavity careful bi-manual exploration of the hepatic ligament is performed to exclude extrahepatic lymph node metastasis. The extrahepatic hilar structures remain untouched if central tumor infiltration or hilus lymph node metastases are excluded.
Liver exploration starts with manual palpation of the complete liver, after having divided the right hepatic triangular ligament.  This is followed by an intraoperative Doppler ultrasonogram to investigate intrahepatic tumor spread and its relation to the main intrahepatic vascular structures, especially the middle hepatic vein, and consequently to determine and mark the transection line.
If a curative procedure appears possible, the right hemi-liver is completely mobilized from the subphrenium and from the retrohepatic inferior vena cava by dividing the posterior liver veins between clips or sutures. This step continues until the right liver vein is free. After isolating the right liver vein, a mersilene loop is inserted for later lifting of the liver during the parenchymal transection.
Next, a retrograde cholecystectomy is performed. The cystic duct is divided between titanium clips.
Subsequently parenchymal dissection is started. The liver is gently held, symmetrically lifted and opened with two towels. The parenchymal transection is performed with a CUSA Dissector (Valleylab, Boulder-CO, USA). Small vessels are sealed with water-irrigated bipolar forceps (Erbe, T bingen, Germany), and larger vessels or bile ducts are divided between ligatures, clips or even sutures. Resections are performed with low central venous pressure CVP (0 5 mm Hg) to reduce blood loss. This phase continues until the intrahepatic right pedicle is completely free. Then, a mersilene loop is inserted to lift the pedicle and allow atraumatic introduction of a vascular stapler (Autosuture TA by Tyco Healthcare Group, Norwalk-Connecticut, USA).
The right pedicle is transected with the stapler and then sharply divided with the knife at its right side.
Then the rest of the parenchymal transection is completed as previously described by suspending the liver using the previously placed mersilene loop.
Subsequently the right hepatic vein is dissected with the vascular stapler and the right liver lobe is removed.
This is followed by careful revision of the cut surface and precise exploration to exclude bile leaks. A white test can be performed at this time if required (7).
The last step is anatomic fixation of the remnant liver lobe by interrupted stitches and then the abdomen is closed by running sutures.
Postoperatively patients are observed on the intensive care unit for one night routinely. If the postoperative course is uneventful with normal sonography and liver function patients are taken to the ward for further recovery.

## Discussion

Hemihepatectomies are mostly performed after preparing the extrahepatic hilar structures within the hepatoduodenal ligament, even in benign tumours or liver metastasis.^1-5^ In various centers, the parenchymal transection is performed under complete or selective hilar vascular occlusion. Major disadvantages of these procedures are the complex preparation of the hilus with the risk of injuring contralateral structures and the possibility of bleeding from portal vein side-branches or impaired perfusion of bile ducts.

This feasible technique of right hemihepatectomy or resection of the left lateral segments with intrahepatic transection of the pedicle leaves the hepatoduodenal ligament completely untouched.

This approach can be used in those liver tumours, where lymphadenectomy is oncologically unnecessary, and in benign indications.
